# Statistical analysis plan for a cluster-randomised trial assessing the effectiveness of implementation of a bedside evidence-based checklist for clinical management of brain-dead potential organ donors in intensive care units: DONORS (Donation Network to Optimise Organ Recovery Study)

**DOI:** 10.1186/s13063-020-04457-1

**Published:** 2020-06-17

**Authors:** Natalia Elis Giordani, Caroline Cabral Robinson, Glauco Adrieno Westphal, Regis Goulart Rosa, Daniel Sganzerla, Alexandre Biasi Cavalcanti, Flávia Ribeiro Machado, Luciano Cesar Pontes Azevedo, Fernando Augusto Bozza, Cassiano Teixeira, Joel de Andrade, Cristiano Augusto Franke, Cátia Moreira Guterres, Itiana Cardoso Madalena, Adriane Isabel Rohden, Sabrina Souza da Silva, Luiza Vitelo Andrighetto, Gabriela Soares Rech, Bruna dos Passos Gimenes, Luciano Serpa Hammes, Daniela Ferreira Salomão Pontes, Maureen O. Meade, Maicon Falavigna

**Affiliations:** 1 Research Projects Office, Hospital Moinhos de Vento (HMV), Rua Ramiro Barcelos, 910, Porto Alegre, RS 90035-001 Brazil; 2 Postgraduate Programme in Epidemiology, Universidade Federal do Rio Grande do Sul (UFRGS), Porto Alegre, RS Brazil; 3 Hospital Municipal São José, Joinville, SC Brazil; 4 Centro Hospitalar Unimed, Joinville, SC Brazil; 5 Adult Intensive Care Unit, HMV, Porto Alegre, RS Brazil; 6 Research Institute, Hospital do Coração (HCor), São Paulo, SP Brazil; 7 Department of Anaesthesiology, Pain and Intensive Care, Universidade Federal de São Paulo (UNIFESP), São Paulo, SP Brazil; 8 Intensive Care Unit, Hospital Sírio-Libanês, São Paulo, SP Brazil; 9 Department of Critical Care and Postgraduate Programme in Translational Medicine, D’Or Institute for Research and Education, Rio de Janeiro, RJ Brazil; 10 Evandro Chagas National Institute of Infectious Diseases, Fundação Oswaldo Cruz (FIOCRUZ), Rio de Janeiro, RJ Brazil; 11 Department of Internal Medicine and Postgraduate Programme in Rehabilitation Science, Universidade Federal de Ciências da Saúde de Porto Alegre (UFCSPA), Porto Alegre, RS Brazil; 12 Organ Procurement Organisation of Santa Catarina (OPO/SC), Florianópolis, SC Brazil; 13 Adult Intensive Care Unit, Hospital de Clínicas de Porto Alegre (HCPA), Porto Alegre, RS Brazil; 14 Trauma Intensive Care Unit, Hospital de Pronto de Socorro (HPS), Porto Alegre, RS Brazil; 15 Office of the Superintendent, HMV, Porto Alegre, RS Brazil; 16 General Coordination Office of the National Transplant System, Ministério da Saúde, Brasília, DF Brazil; 17 Department of Medicine and Department of Health Research Methods, Evidence and Impact, McMaster University, Hamilton, Ontario Canada

**Keywords:** Organ donor, Organ transplantation, Donor management, Donor management goals, Checklist

## Abstract

**Background:**

The quality of clinical care of brain-dead potential organ donors may help reduce donor losses caused by irreversible or unreversed cardiac arrest and increase the number of organs donated. We sought to determine whether an evidence-based, goal-directed checklist for donor management in intensive care units (ICUs) can reduce donor losses to cardiac arrest.

**Methods/design:**

The DONORS study is a multicentre, cluster-randomised controlled trial with a 1:1 allocation ratio designed to compare an intervention group (goal-directed checklist for brain-dead potential organ donor management) with a control group (standard ICU care). The primary outcome is loss of potential donors due to cardiac arrest. Secondary outcomes are the number of actual organ donors and the number of solid organs recovered per actual donor. Exploratory outcomes include the achievement of relevant clinical goals during the management of brain-dead potential organ donors. The present statistical analysis plan (SAP) describes all primary statistical procedures that will be used to evaluate the results and perform exploratory and sensitivity analyses of the trial.

**Discussion:**

The SAP of the DONORS study aims to describe its analytic procedures, enhancing the transparency of the study. At the moment of SAP subsmission, 63 institutions have been randomised and were enrolling study participants. Thus, the analyses reported herein have been defined before the end of the study recruitment and database locking.

**Trial registration:**

ClinicalTrials.gov, NCT03179020. Registered on 7 June 2017.

## Background

Proper management of brain-dead potential organ donors is essential to increase the number and quality of donated organs. In this setting, systematisation of care through a checklist of clinical goals can help reduce the mismatch between organ supply and demand. The role of checklists in healthcare, particularly to help prevent omissions in complex procedures and settings, is now well established. Previous studies have shown that use of goal-directed checklists can contribute to the systematic application of clinical practice guidelines, leading to greater adherence to evidence-based interventions and improving clinical outcomes [1–7]. However, some before-and-after studies have failed to demonstrate consistent benefit of checklist application in processes of care and patient safety [7–9]. Additionally, the current evidence cannot support the use of a goal-directed checklists in the management of brain-dead potential organ donors, given the small number of studies, their relatively high risk of bias, and the inconsistency of their findings [10–17]. DONORS (Donation Network to Optimise Organ Recovery Study) aims to address this gap by evaluating the effectiveness of implementation of an evidence-based bedside checklist containing goals and recommendations for the management of brain-dead potential organ donors in reducing potential donor losses to cardiac arrest, thus increasing the number of actual organ donors and the number of organs recovered per actual donor. To ensure that analyses are performed in compliance with good clinical practice and to minimise the risk of bias, this statistical analysis plan (SAP) describes the analytical objectives and procedures of the trial before the end of recruitment and database locking.

## Trial overview

DONORS is a parallel cluster-randomised trial designed to assess the effectiveness of implementation of an evidence-based bedside checklist containing goals and recommendations for clinical management of brain-dead potential organ donors in the adult intensive care unit (ICU). A detailed description of the study background, rationale, design, and eligibility criteria, as well as a description of the intervention and co-intervention, has been published elsewhere [18].

For sample size determination, we considered that with 60 ICUs, we will need to include 19 brain-dead potential organ donors per site (1140 potential donors) to detect an absolute reduction of donor losses due to cardiac arrests of 10% (from 28% in the control group to 18% in the intervention group) [11], considering an intraclass correlation coefficient of 0.05, power of 80%, and a two-sided alpha level of 5%. Therefore, considering a possible variation in cluster size and its impact on statistical power, we intend to include a minimum of 60 ICUs with at least 1200 potential organ donors, not allowing more than 30 participants in each cluster. WinPepi software version 11.65 (http://www.brixtonhealth.com/pepi4windows.html) and the StatsToDo website (www.statstodo.com/index.php) were used for sample size determination.

ICUs in Brazilian hospitals with an average of at least ten annual notifications of potential organ donors in the 2 years preceding study selection were randomly allocated to the intervention or control group in a 1:1 ratio using blocks of variable sizes (2 and 4), then stratified dichotomously by the estimated median number of annual notifications of brain death (≤ 29 vs. > 29 notifications). Consecutive brain-dead potential organ donors (as confirmed by the first clinical examination consistent with brain death) aged 14–90 years will be screened. Only patients already in the ICU or admitted to the ICU within 3 h of initial assessment for brain death are included. A timeline cluster diagram [19] depicting how to improve transparency with respect to the study’s risk of bias is provided in Additional file 1. Timeline cluster diagram.

A checklist for management of brain-dead potential organ donors will be applied to the intervention group. The checklist was designed to address goals and recommendations from clinical practice guidelines that involve temperature management, mechanical ventilation, haemodynamics, endocrine and metabolic management (including administration of vasopressin and/or desmopressin, hydrocortisone, and insulin), and administration of antibiotics and blood products as required. The checklist should be applied immediately after potential donor inclusion in the study and every 6 h thereafter until organ recovery or loss of the potential donor due to irreversible cardiac arrest (not reversed despite resuscitation manoeuvres) or unreversed cardiac arrest (resuscitation manoeuvres were not implemented), family refusal, or a contraindication to organ donation arising after patient inclusion [18]. ICUs assigned to the control group will continue to provide standard care to potential organ donors, without access to the goal-directed checklist [18].

With the aims of standardising family interviews for organ donation and reducing variability between study sites, we planned a co-intervention, which consists of training one member of each ICU staff and one member of each intra-hospital transplant co-ordination (IHTC) committee in family interviews for organ donation. The training consists of a face-to-face course with theoretical and practical activities, as well as an online course provided for all ICU staff members and IHTC members from all centres.

The primary outcome is the number of brain-dead potential organ donors lost to cardiac arrest. The following are secondary outcomes: the number of actual organ donors and the number of solid organs recovered per actual donor. The following are exploratory outcomes: (1) the proportion of potential donors with adequate respiratory parameters, (2) the proportion of potential donors with adequate body temperature, (3) the proportion of potential donors with adequate circulatory parameters, and (4) organ dysfunction score assessed by the Sequential Organ Failure Assessment (SOFA). In addition, eight other outcomes related to goal adherence will be measured.

## Statistical analysis plan

### Overall principles

The main analysis for each outcome will be performed at the subject level. All participants will be included in the analysis and will be analysed according to their allocated treatment group (control or intervention), regardless of the extent of adherence to protocol (intention to treat). Furthermore, all analyses will account for the cluster-randomised design, thus ensuring correct type I error rates and confidence intervals. A significance level of 0.05, adjusted for multiplicity as appropriate, will be adopted for all comparisons. Analysis will start once all data have been obtained from the last included patient, the database has been cleaned and locked, and the plan has been submitted for publication. All analyses will be performed in the R software environment (R Foundation for Statistical Computing) [20].

### Handling of missing data

We anticipate minimal missing values for exploratory outcomes, given that the study procedures involve both training of site research staff and independent remote and on-site data monitoring by the study coordinator. Nevertheless, the coordinating centre will contact site investigators to retrieve any missing data values. Analyses for primary and secondary outcomes will be based on participants for whom outcome data are available (that is, available case analysis). Thus, no imputation will be performed for primary or secondary outcomes, except in the context of sensitivity analysis for the primary outcome. Additional details about the simulated outcomes are given in the “Sensitivity analyses” section below.

For exploratory outcomes, we will perform data imputation only for the SOFA score components. Imputation rules will be followed in the order below:

1. For all SOFA components, we will impute the information from the latest available time point of assessment.
2. For the respiratory component, if the partial oxygen pressure (PaO_2_)/fraction of inspired oxygen (FIO_2_) ratio is not available, we will estimate this score on the basis of peripheral oxygen saturation (SaO_2_)/FIO_2_ ratio adjusted to the positive end-expiratory pressure (PEEP), according to a previously validated method [21].
3. For the coagulation, liver, and renal components, if there are missing values at any point during follow-up, we will impute a score of 0 (corresponding to normality), except if pre-existing comorbidities are present (participants with renal impairment requiring dialysis will be imputed a score of 4 and participants with liver cirrhosis a score of 2) [22].

### Definition of analysis sets

At the cluster level, the population includes all randomised ICUs that recruited at least one brain-dead potential organ donor. At the subject level, the population includes up to 30 consecutive brain-dead potential donors in each cluster, regardless of protocol deviations. This includes potential donors allocated to the intervention group whose management was performed without application of the proposed intervention, as well as potential donors randomised to the control group whose management included some sort of checklist for the management of organ donors.

### Statistical analyses

#### Flow of participants

The flow of participants is displayed in a detailed diagram that meets the criteria of the Consolidated Standards of Reporting Trials (CONSORT) 2010 extension for cluster-randomised trials [23]. This flow diagram has been published elsewhere [18]. The description will include information on eligibility criteria and loss to follow-up at both the cluster and subject levels.

#### Adherence to study intervention

The checklist consists of 13 goals and 14 actions for management of brain-dead potential organ donors. We will classify adherence to each specific action as complete if (a) the recommended course of action was performed or (b) there was no need for action according to the checklist. Adherence to each individual component will be presented as a proportion, considering the ratio between the total number of actions adhered to and the total number of potential actions to be performed. Estimation of adherence will consider the checklists applied at baseline, 6 h, 12 h, 24 h, 48 h, 72 h, 96 h, 120 h, 144 h, and 168 h as available.

#### Baseline characteristics

The baseline characteristics of all participants, stratified by study arm, are presented in Table 1, without statistical comparisons between groups (to avoid unnecessary testing). Continuous variables will be presented as mean and standard deviation (SD) or median and interquartile range (IQR), as appropriate. Categorical variables will be presented as absolute (*n*) and relative (%) frequencies.

**Table 1:** Baseline characteristics of study participants

	Intervention arm	Control arm
ICU characteristics		
Number of hospital beds, central tendency (dispersion), n	xx.x (xx.x)	xx.x (xx.x)
Number of ICU beds, central tendency (dispersion), n	xx.x (xx.x)	xx.x (xx.x)
Number of ICU beds/hospital beds, central tendency (dispersion), n	xx.x (xx.x)	xx.x (xx.x)
Type of ICU		
Surgical, n/total (%)	xx/xx (xx.x)	xx/xx (xx.x)
Medical, n/total (%)	xx/xx (xx.x)	xx/xx (xx.x)
Mixed, n/total (%)	xx/xx (xx.x)	xx/xx (xx.x)
Hospital type		
Public, n/total (%)	xx/xx (xx.x)	xx/xx (xx.x)
Private, n/total (%)	xx/xx (xx.x)	xx/xx (xx.x)
Teaching activity, n/total (%)	xx/xx (xx.x)	xx/xx (xx.x)
Transplant centre, n/total (%)	xx/xx (xx.x)	xx/xx (xx.x)
Number of brain death notifications per year,^a^ central tendency (dispersion), [n]	xx.x (xx.x)	xx.x (xx.x)
Participant characteristics		
Age in years, central tendency (dispersion), [n]	xx.x (xx.x)	xx.x (xx.x)
Age > 60 years, n/total (%)	xx/xx (xx.x)	xx/xx (xx.x)
Female sex, n/total (%)	xx/xx (xx.x)	xx/xx (xx.x)
Male sex, n/total (%)	xx/xx (xx.x)	xx/xx (xx.x)
SAPS 3 score at ICU admission, central tendency (dispersion), [n]	xx.x (xx.x)	xx.x (xx.x)
Comorbidities		
Diabetes mellitus, n/total (%)	xx/xx (xx.x)	xx/xx (xx.x)
Hypertension, n/total (%)	xx/xx (xx.x)	xx/xx (xx.x)
Dialytic renal failure, n/total (%)	xx/xx (xx.x)	xx/xx (xx.x)
Chronic respiratory disease, n/total (%)	xx/xx (xx.x)	xx/xx (xx.x)
Heart failure, n/total (%)	xx/xx (xx.x)	xx/xx (xx.x)
Chronic liver disease, n/total (%)	xx/xx (xx.x)	xx/xx (xx.x)
Cause of brain injury		
Trauma, n/total (%)	xx/xx (xx.x)	xx/xx (xx.x)
Stroke, n/total (%)	xx/xx (xx.x)	xx/xx (xx.x)
Anoxia, n/total (%)	xx/xx (xx.x)	xx/xx (xx.x)
Other, n/total (%)	xx/xx (xx.x)	xx/xx (xx.x)
SOFA score at enrolment, central tendency (dispersion), [n]	xx.x (xx.x)	xx.x (xx.x)
Use of antimicrobial medication,^b^ n/total (%)	xx/xx (xx.x)	xx/xx (xx.x)
Length of hospital stay in days before brain death diagnosis, central tendency (dispersion), [n]	xx.x (xx.x)	xx.x (xx.x)

*SAPS* Simplified Acute Physiology Score, *SOFA* Sequential Organ Failure Assessment

Chronic liver disease is defined as biopsy-proven cirrhosis or proven portal hypertension or previous history of hepatic insufficiency, encephalopathy, or coma. Chronic respiratory disease is defined as restrictive, obstructive, or vascular disease severe enough to limit performance of the activities of daily living or chronic hypoxia, hypercapnia, polycythaemia, pulmonary hypertension, or ventilator dependence

^a^ Number of brain death notifications per year considers the percentage of brain-dead potential organ donors clinically managed in the intensive care unit

^b^Identified at the time of first clinical examination

#### Primary outcome

The primary outcome is loss of brain-dead potential organ donors to cardiac arrest (defined as any loss from irreversible or unreversed cardiac arrest that occurs after patient enrolment while the potential donor remains eligible for organ donation, i.e., with no contraindications and after family approval or with family decision pending). Data will be recorded up to 14 days after participant enrolment. Loss to cardiac arrest occurring after family refusal for organ donation or detection of a contraindication to donation will not be recorded as a primary outcome. To assess the effect of study interventions on loss due to cardiac arrest, we will use survival analysis adjusted for cluster effect (frailty model) [24]. Participants will be considered at risk for occurrence of the outcome of interest only while under consideration as brain-dead potential organ donors. Thus, data will be censored in the following circumstances: (a) family refusal, (b) contraindications to organ donation, or (c) organ retrieval. Results will be presented as hazard ratio (HR) and 95% confidence interval (CI). Model assumptions (e.g., proportional hazards and residuals analysis) will be assessed using appropriate tests and plots (Table 2).

**Table 2:** Primary and secondary study outcomes

Outcomes	Intervention arm	Control arm	Type of effect estimate	Effect estimate (CI)	***P*** value^**a**^
Primary					
Potential organ donors lost due to cardiac arrest, n/total (%)^b^	x/x (xx.x)	x/x (xx.x)	HR	x.xx (x.xx-x.xx)^c^	x.xx
Secondary					
Actual organ donors, n/total (%)	x/x (xx.x)	x/x (xx.x)	RR RD	x.xx (x.xx-x.xx)^d^ x.xx (x.xx-x.xx)^d^	x.xx
Organs recovered per actual donor, central tendency (dispersion)	xx.x (xx.x)	xx.x (xx.x)	MD	x.xx (x.xx-x.xx)^d^	x.xx

*Abbreviations: CI* Confidence interval, *HR* Hazard ratio, *MD* Mean difference *RD* Risk difference, *RR* Risk ratio

^a^Adjusted for multiple comparisons with Bonferroni correction when appropriate

^b^Intracluster correlation coefficient

^c^95% confidence interval

^d^97.5% confidence interval

##### Sensitivity analyses

We will conduct sensitivity analysis of the primary outcome adjusted for (1) adherence to the intervention, (2) time elapsed between first clinical examination consistent with brain death and inclusion in the study, (3) occurrence of possible failures in the screening and inclusion of consecutive participants, (4) estimated number of brain death notifications in each ICU (≤ 29 vs. > 29, according to the stratification variable), and (5) donation rate for each centre before the study.

For analyses 1 and 2, the cut-off will be the median observed across all sites. For analysis 3, in order to estimate the number of recruitment failures per site, we will consider the total number of brain death notifications according to the Brazilian National Transplant System records during the study period. The outcome will be simulated considering a binomial distribution with probability of success equal to the percentage of losses of brain-dead potential organ donors due to cardiac arrest observed in the control group. The total follow-up time for each recruitment failure will be simulated considering the minimum, median, and maximum values observed in the sites, according to the observed simulated outcome. Analysis 4 will consider the dichotomous stratification used for random allocation, based on the estimated annual number of notifications of brain death in each site (≤ 29 vs. > 29). Analysis 5 will consider the donation rate for each centre for the year 2016, which is available in the Brazilian National Transplant System records.

##### Subgroup analyses

There will be three subgroup analyses defined for the primary outcome, considering the variables age > 60 years, cause of the insult leading to potential brain death (traumatic vs. non-traumatic), and patient severity upon ICU admission defined by the Simplified Acute Physiology Score 3 (SAPS 3; cut-off will be established regarding the overall median score). The consistency of intervention effects across the mentioned subgroups will be assessed by tests of interaction. The Bonferroni correction will be applied to adjust the multiple subgroup analysis. We will perform three tests, so the critical alpha will be 0.017.

#### Secondary outcomes

The following statistical procedures will be used to evaluate the secondary outcomes of interest:

1. Number of actual organ donors, indexed to brain-dead potential donors (proportion): Differences in actual donor ratios between the intervention and control groups will be analysed using generalised estimating equations (GEE) [25–28], with the appropriate distribution and adjustment for cluster effect (Poisson distribution with log-link function for estimation of risk ratio [RR] and Poisson distribution with identity link function for estimation of risk difference [RD] [26]). This outcome will be presented as RR and RD with 97.5% CI, adjusted for multiple comparisons by Bonferroni correction. We will conduct a sensitivity analysis considering the number of kidneys harvested.
2. Number of solid organs recovered per actual donor (from zero to seven organs per donor, as follows: liver, heart, pancreas, two lungs, and two kidneys): Between-group differences in the mean number of solid organs recovered per actual donor will be compared using GEE with the appropriate distribution and adjustment for cluster effect (Poisson distribution with identity link function). This outcome will be presented as mean difference (MD) and 97.5% CI adjusted for multiple comparisons with Bonferroni correction.

The Bonferroni correction will be used to adjust the analyses of secondary outcomes for multiplicity, considering two comparisons of interest; thus, the critical alpha will be 0.025. The secondary outcomes will be presented as shown in Table 2.

#### Exploratory outcomes

We will analyse the following exploratory outcomes [18], for which no adjustment for multiple comparisons will be made:

1. Proportion of potential donors with adequate respiratory parameters, defined as PaO_2_/FIO_2_ ratio ≥ 200. In the absence of PaO_2_ and FIO_2_ parameters measured simultaneously, adequate respiration will be defined as SaO_2_/FIO_2_ ≥ 240 (if PEEP < 8), ≥ 259 (if PEEP 8–12), or ≥ 234 (if PEEP > 12) [21] (will be presented as RR and 95% CI).
2. Proportion of potential donors with adequate body temperature, defined as 34–35 °C if haemodynamically stable and > 35 °C if mean arterial pressure (MAP) < 65 mmHg or noradrenaline or dopamine is required (will be presented as RR and 95% CI).
3. Proportion of potential donors with adequate circulatory parameters, considering the following as inadequate: MAP < 65 mmHg or noradrenaline ≥ 0.1 μg/kg/min or dopamine ≥15 μg/kg/min (will be presented as RR and 95% CI).
4. SOFA score, as per Vincent et al. (1996) [22] (will be presented as MD and 95% CI).

We will also analyse data for additional exploratory outcomes related to goal adherence:

1. Proportion of potential donors receiving protective ventilation: tidal volume (Vt) 6–8 ml/kg of predicted body weight and PEEP ≥ 8 cmH_2_O (will be presented as RR and 95% CI).
2. Proportion of potential donors receiving vasopressin if on noradrenaline or dopamine (will be presented as RR and 95% CI).
3. Proportion of potential donors receiving hydrocortisone if on noradrenaline or dopamine (will be presented as RR and 95% CI).
4. Proportion of potential donors with Na < 155 mEq/L (will be presented as RR and 95% CI).
5. Proportion of potential donors with Mg > 1.6 mEq/L (will be presented as RR and 95% CI).
6. Proportion of potential donors with K 3.5–5.5 mEq/L (will be presented as RR and 95% CI).
7. Proportion of potential donors with capillary blood glucose < 180 mg/dl (will be presented as RR and 95% CI).
8. Proportion of potential donors receiving antibiotics (among those with infection). Will be presented as RR and 95% CI.

All exploratory outcomes will be analysed using a repeated-measures GEE considering each 24-h fraction of the follow-up period (Poisson distribution with log-link function for outcomes 1, 2, 3, and 5 to 12 and normal distribution with identity link function for outcome 4), with exception of exploratory outcome number 12.

### Differences between the study protocol and statistical analysis plan

We included the following sensitivity analysis for the primary outcome of the study:

- Occurrence of potential failures in the screening and inclusion of consecutive participants
- Estimated number of brain death notifications in ICU
- Donation rate for each centre before the study

We renamed tertiary outcomes as exploratory outcomes, and we also included additional exploratory outcomes (numbers 5 to 12 as described in the “Exploratory outcomes” subsection above).

## Discussion and trial status

In this SAP, we have presented the statistical procedures that will allow assessment of the effectiveness of implementation of a bedside evidence-based checklist for clinical management of brain-dead potential organ donors in intensive care units, as compared with usual management (standard of care, without checklist support). The SAP was concluded without any knowledge by the authors about the database or results of the trial. Currently, 63 institutions have been randomised, and 1535 brain-dead potential organ donors have been included in the study. The list of sites is available on the ClinicalTrials.gov website (identifier NCT03179020) and in the protocol publication [18]. We are auditing the data (allowing for correction of inconsistencies and missing data) and we expect to lock the database by July, 2020.

## Supplementary information

**Additional file 1. ** Timeline cluster diagram.

## Data Availability

The datasets generated and/or analysed during the current study are not publicly available, but are available from the corresponding author on reasonable request and with permission of the Brazilian Ministry of Health.

## Abbreviations

CI: Confidence interval
CONSORT: Consolidated Standards of Reporting Trials
DONORS: Donation Network to Optimise Organ Recovery Study
FIO_2_: Fraction of inspired oxygen
GEE: Generalised estimating equations
HR: Hazard ratio
ICU: Intensive care unit
IHTC: Intra-hospital transplant co-ordination
IQR: Interquartile range
MAP: Mean arterial pressure
MD: Mean difference
NCT: Number of clinical trials
PaO_2_: Partial oxygen pressure
PEEP: Positive end-expiratory pressure
RD: Risk difference
RR: Risk ratio
SaO_2_: Peripheral oxygen saturation
SAP: Statistical analysis plan
SAPS: Simplified Acute Physiology Score
SD: Standard deviation
SOFA: Sequential Organ Failure Assessment
Vt: Tidal volume
