# Supplementary figures and images for: Statistical analysis plan for a cluster-randomised trial assessing the effectiveness of implementation of a bedside evidence-based checklist for clinical management of brain-dead potential organ donors in intensive care units: DONORS (Donation Network to Optimise Organ Recovery Study)
Source: Trials. 2020 Jun 17;21:540. doi: 10.1186/s13063-020-04457-1 (PMC7298918; doi:10.1186/s13063-020-04457-1)

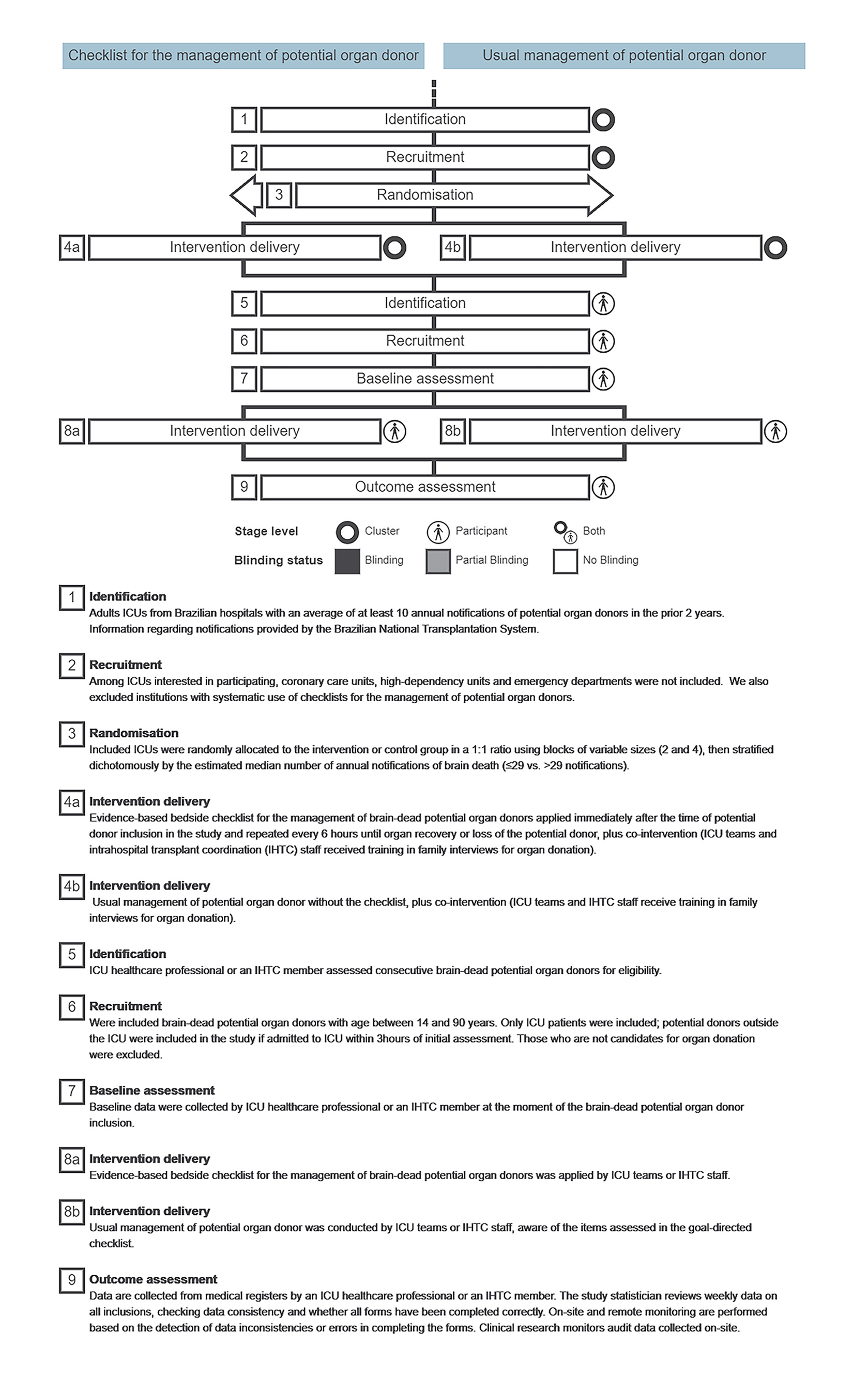

Supplement: Supplementary file 1 — Additional file 1. Timeline cluster diagram. [file 13063_2020_4457_MOESM1_ESM.jpg]
